# Intelligent Packaging in the Food Sector: A Brief Overview

**DOI:** 10.3390/foods8010016

**Published:** 2019-01-07

**Authors:** Patricia Müller, Markus Schmid

**Affiliations:** Faculty of Life Sciences, Albstadt-Sigmaringen University, Anton-Günther-Str. 51, 72488 Sigmaringen, Germany; muellepa@hs-albsig.de

**Keywords:** packaging, intelligent packaging, smart packaging, indicators, sensors

## Abstract

The trend towards sustainability, improved product safety, and high-quality standards are important in all areas of life sciences. In order to satisfy these requirements, intelligent packaging is used in the food sector. These systems can monitor permanently the quality status of a product and share the information with the customer. In this way, food waste can be reduced and customer satisfaction can be optimized. Depending on the product, different types of intelligent packaging technologies are used and discussed in this review. The three main groups are: data carriers, indicators, and sensors. At this time, they are not that widespread, but their potential is already known. In which areas intelligent packaging should be implemented, how the systems work, and which values they offer are dealt in this review.

## 1. Introduction

**Figure 1:**
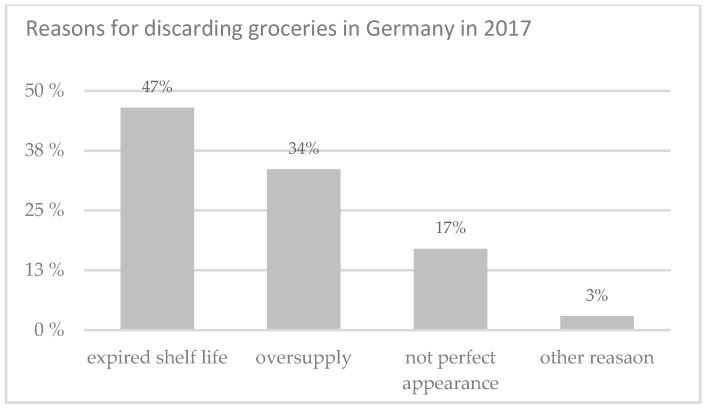
Reasons for discarding groceries in Germany in 2017, adapted from [7].

Packaging separates the products from the external environment and has in general four basic functions: protection, communication, convenience, and containment [1]. It communicates with the consumer by written texts or graphics and simplifies the handling of the contained products with practical features such as reclose- or microwave-ability. Furthermore, it offers differently shaped and sized containers and adapts to the customer’s lifestyle [2]. In addition to improved marketing and distribution, packaging also slows quality decay. That is why they contribute significantly to safe delivery and preservation of packaged food [3]. However, a complete elimination of the quality loss is not possible. Intrinsic properties of highly perishable foods change after processing. This can lead to an increase in quality (e.g., the ripening of fruits to a certain level) or to a quality loss: Depending on the contents of the package, biological, chemical, or physical processes occur, which ultimately lead to spoilage of the product [4,5]. These changes are in most cases difficult to assess by the consumers. In fear groceries could be spoiled, many consumers throw products away which would actually have still been suitable for consumption. Often a small deviation from the norm, either the color, the consistency, or even the passing of the best before date leads to products ending up in the bin [6,7] (see Figure 1). 

**Figure 2:**
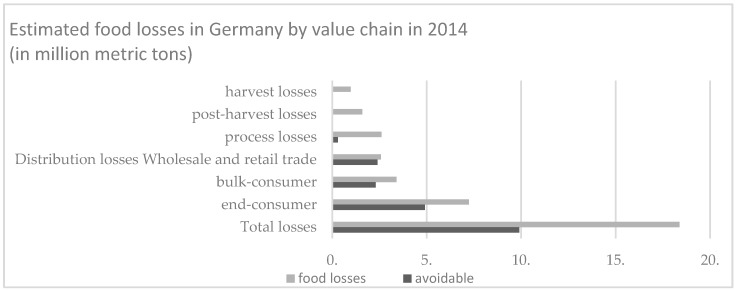
Estimated food losses in Germany by value chain in 2014, adapted from [8].

If we compare the loss of food within the value chain, it is the end consumer who contributes most. What is even worse is that much of the food loss could be avoided (see Figure 2) [8].

To reduce this unintentional product waste, so-called intelligent packaging concepts could be utilized. But this is not the only advantage these technologies provoke [9]. 

Microbiological and chemical tests of the products are regularly performed at the company level during production and before delivery [10]. But in most cases there is no such control after delivery to the supermarket. Intelligent packaging will close this gap as they are able to monitor and display the quality status from the point of manufacture up to the customer. This permanent monitoring not only minimizes unnecessary food waste but also protects consumers against potential food poisoning, maximizes the efficiency of the food industries, and improves traceability [11,12].

Preserving food quality is also an important area of research, as it is directly related to the global aim of improving the quality of our lives. Furthermore, there is a growing demand by consumers due quality and safety properties. These issues are highly dependent on the applied packaging materials [13]. Intelligent Packaging has potential to improve product safety, reduce environmental impact and increase the attractiveness of the packaged product and also of the food companies [12,14,15].

## 2. Overview of Intelligent Packaging Technologies and Concepts

### 2.1. Definition and Legal Framework

The EFSA defines intelligent packaging materials as “materials and articles that monitor the condition of packaged food or the environment surrounding the food” [16]. They have the ability to communicate the conditions of the packaged product, but they do not interact with the product [17]. Their aim is to monitor the product and transmit the information to the consumers. This can be information about the condition of a package and its contents, time of manufacture or storage conditions [18]. Depending whether it is a simple or a reactive intelligent packaging, these can be placed on the primary (outside or inside), secondary or tertiary packaging [15].

In order that the intelligent packaging can be used for commercial purposes, legal requirements must be fulfilled. For many years, there was no legal framework for intelligent packaging in the EU. That is why the systems are not widespread as they are in the USA, Australia or Japan [19]. Furthermore, due to the stricter laws in the EU, intelligent packaging from the USA cannot be introduced so easily in Europe [20].

Nowadays general requirements are dealt with in Regulation EC No 1935/2004 on materials and articles intended to come into contact with food. Regulation EU No 10/2011 deals in particular with plastic materials and articles. Article 3 of EC No 1935/2004 commands that intelligent packaging should not transfer their constitutions to food in quantities. This could endanger human health, bring about an unacceptable change in the composition of the food or bring about a deterioration in the organoleptic characteristics thereof. Article 4 regulates on the one hand the labelling, which must indicate that the parts are non-edible and the packaging is intelligent. On the other hand, it requires that the transmitted information through intelligent packaging does not mislead the consumer [21].

Rules on good manufacturing practice for materials and articles that are permitted to come into contact with food are also described in Regulation EC No 2023/2006 [22].

The Regulation EC No 450/2009 deals in more detail with the requirements and approval of active and intelligent materials and articles intended to come into contact with food [23].

### 2.2. Different Types and Concepts of Intelligent Packaging

In general there are three main technologies which are used for intelligent packaging systems: data carriers, indicators, and sensors [10]. A subdivision according to the following types is also possible:

- Environmental conditions: This species monitors conditions which can lead to changes in the quality characteristics of the food. Examples of these types are time temperature indicators, gas leakage indicators, and relative humidity sensors. Depending on the monitoring factor, these systems can be placed outside or inside the packaging.
- Quality characteristics or quality indicator compounds: This type is used for the direct monitoring of the quality attributes of the food itself. Examples are bio sensors and freshness sensor/indicators. These devices are usually located inside the packaging.
- Data carriers: These systems are only used to store and transfer data, while indicators and sensors are used to monitor the external environment and display the information afterwards [4,15].

#### 2.2.1. Data Carriers

Data carriers help to make the information flow within the supply chain more efficient. The function of data carriers is not to monitor the quality of the products, but to guarantee traceability, automatization, theft protection, or counterfeit protection [24]. To ensure this, data carriers store and transmit information about storage, distribution, and other parameters. Therefore, they are often placed on tertiary packaging. The most frequently used data carriers are barcode labels and RFID (Radio Frequency Identification) tags [10].

##### Barcodes

Barcodes are cheap, easy to use, and are widely used to facilitate inventory control, stock recording, and checkout [25]. In general, barcodes can be divided into one-dimensional and two-dimensional ones. Depending on the type, they have different storage capacities [10].

**Figure 3:**
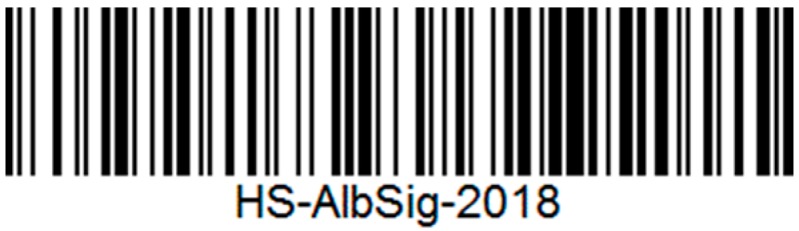
Barcode.

A one-dimensional barcode is a pattern of parallel spaces and bars (see Figure 3). The different arrangement of the bars and gaps results in the coding of data. A barcode scanner and an associated system can translate the coded information [10].

**Figure 4:**
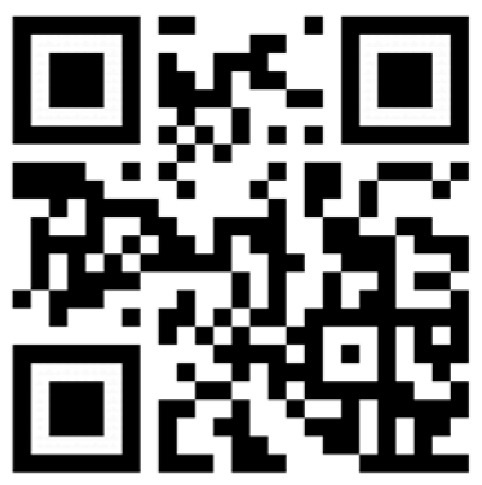
QR-Code.

Two-dimensional barcodes offer more memory capacity (e.g., for packaging date, batch number, packaging weight, nutritional information, or preparation instructions) because of the combination of dots and spaces arranged in an array or a matrix. This provides great convenience for retailers and consumers. An example of 2D barcodes are QR (quick response) Codes (see Figure 4) [14].

##### Radio Frequency Identification (RFID) Technology

RFID tags are advanced data carrier with a data storage up to 1 MB, as well as non-contact and non-line-of-sight ability to collect real time data. These collect, store and transmit real-time information to a user’s information system. In comparison to barcodes, RFID tags are more expensive and need a more powerful electronic information network [14]. On the other side, the information can be loaded electronically on these tags and can be changed again [26]. Furthermore, RFID offers further advantages for the entire food supply chain. These include traceability, inventory management and promotion of quality and safety [27].

**Figure 5:**
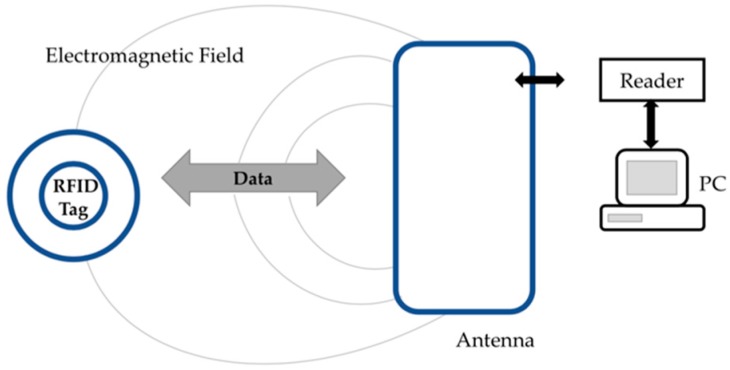
The working principle of radio frequency identification (RFID) tag, adapted from [28].

A RFID system consists of three compounds: a tag formed by a microchip connected to a tiny antenna, a reader that emits radio signals and receives answers from the tag in return and a middleware that bridges the RFID hardware and enterprise applications (see Figure 5) [27,28]. 

##### Time Temperature Indicator (TTI) Integrated Barcodes and RFID Tags

Barcodes and QR Codes are the first so-called intelligent packaging technologies. Meanwhile they have been further developed and integrated into TTIs. The principle is based on the fact that a label is scanned and information about the product as well as the temperature progression is given. Compared to traditional data carriers, these systems can not only be used to track the distribution chain, but can also help reduce food waste [29].

For example, Bioett has a TTI-Barcode system on the market where the data is captured with a portable scanner, displayed on a computer monitor and downloaded to a database for analysis. Infratab has developed a battery powered TTI-RFID tag that uses a microchip to capture the temperature progression to determine the shelf life of a product. A biosensor-barcode, called Food Sentinel System, was developed by SIRA Technologies. A specific pathogenic antibody is bonded to a membrane-forming part of the barcode. If bacteria are present, a dark bar is formed that makes the barcode unreadable when scanning [1].

#### 2.2.2. Indicators

Indicators determine the presence or absence of a substance, the extent of a reaction between different substances or the concentration of a particular substance. This information is visualized by direct changes, e.g., different color intensities [28]. Depending on the indicator they are placed inside or outside of the package [10].

##### Time Temperature Indicators (TTIs)

Temperature is an important factor in determining the shelf life of a food product. Deviations in the temperature profile can result in growth or survival of microorganisms, which ultimately causes in spoilage of the product. Furthermore, incorrect freezing can denature the proteins of meat or other products. Whether the cold chain or a required temperature is properly maintained during the food supply chain, time temperature indicators can be used [14,30].

In general, time temperature indicators or integrators are simple, inexpensive gadgets attached to the package. Three types can be distinguished: critical temperature indicators, which show if products have been heated above or cooled below a permitted temperature. Secondly, partial history indicators, which indicate if a product has been subjected to temperature, that cause a change in product quality. Thirdly, a full history indicator which records the complete temperature profile along the food supply chain [15,31].

The functional principle of TTIs is based on the detection of time and temperature dependent mechanical, chemical, electrochemical, enzymatic, or microbiological changes of a food product. For example, chemical or physical responses are based on acid–base reactions or polymerization towards time and temperature. In contrast, biological responses are based on biological changes such as microorganisms, spores or enzymes in relation to time and temperature [32]. The measured values are usually expressed as a visible response, like color changes or mechanical deformations [33].

**Figure 6:**
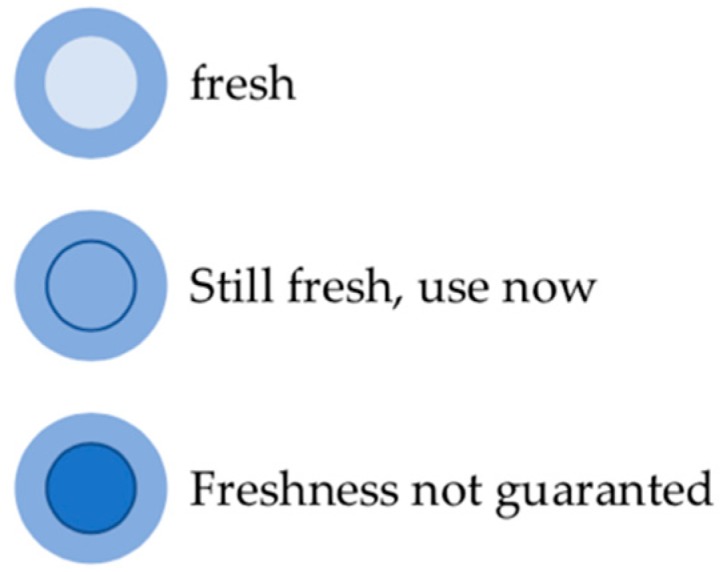
Principle of Lifeline’s Fresh-Check Indicator time temperature indicator (TTI), adapted from [35].

Because of this simple functionality TTIs are recognized as user-friendly and readily usable devices [34]. An example of a TTI indicator is the Fresh-Check from Lifeline technologies. Its function is based on a polymerization reaction resulting in a color change in the indication range. A clear center indicates a fresh TTI. If the color of the active center matches the outer ring, the product should be consumed soon. TTIs of not fresh products have a dark center (see Figure 6) [35].

Further examples of TTIs and their properties are presented in Table 1 [36].

##### Freshness Indicators

Freshness indicators supervise the quality of food products during storage and transportation. Reasons for the loss of freshness can be disadvantageous conditions or exceeded durability. Therefore, they submit information about microbiological growth, presence of microbiological metabolites or chemical changes of the products [39,40]. Quality indicating metabolites are for instance glucose, organic acids, ethanol, volatile nitrogen compounds, biogenic amines, carbon dioxide, ATP degradation products, and sulphuric compounds [14,41]. To be able to be in contact with the compounds the freshness indicators must be placed inside the packaging. Depending on the indicator, this information can be detected by different methods (see Table 2) [36].

**Figure 7:**
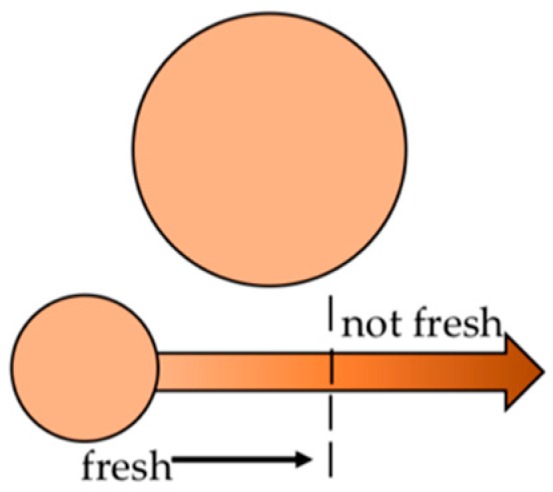
Principle of SensorQ™, Smart Sensor Label by FQSI, adapted from [31].

An example for a freshness indictor is a sensor label from FQSI (Food Quality Sensor International Inc., Lexington, MA, USA), which is able to detect biogenic amines. The SensorQ™ sticker ticket is applied on the inside of the packaging and indicates that a critical level of bacterial growth has been reached by a change of color (orange to brown) (see Figure 7) [31]. The operating principle of biogenic amine sensors is based on amine oxidases or transglutamase. Lactic acid sensors operate on the basis of lactate oxidase and peroxidase activities [36]. Glucose sensors use glucose oxidases that are immobilized on the surface of the electrodes. Glucose oxidase is an enzyme that catalyzes the oxidation of glucose [43].

##### Gas Indicators

Gas indicators indicate the quality condition of the food depending on the indoor atmosphere. A sensor detects and reacts to changes in the atmosphere inside the packaging, while the actual indicator displays the quality status. The modifications of the atmosphere are based on the one hand of the food activity, such as enzymatic or chemical reactions, and on the other hand of the package nature and environmental conditions, such as gas generation by microorganisms metabolism or gas transmission through the packaging. Most of them monitor the oxygen and carbon dioxide concentrations [42]. But also water vapor, ethanol, hydrogen sulfide, and other gases are checked [14]. The concentrations of these gases often correlate closely with the advance of spoilage [42]. The functionality of most devices is based on redox dyes, a reducing compound and an alkaline component [10]. In order to be able to monitor the gases, indicators must be placed inside the packaging. But many of these indicators have a loss of color due to the moisture in the packaging. However, companies are already researching UV-activated colorimetric indicators [44] that show less dye leaching because of encapsulation or coating technologies [10,45].

#### 2.2.3. Sensors

A sensor is defined as a device used to detect, locate or quantify energy or matter giving a signal for the detection or measurement of a physical or chemical property to which the device responds [46].

Most sensors consist of two components: They have a sensor part which is also known as the receptor. This can detect the presence, activity, composition or concentration of certain chemical or physical analytes. The physical or chemical information is also converted by the receptor into a form of energy that can be measured by the second component, the transducer [47]. Furthermore, the transducer is used to convert the measured signal into a useful analytic signal. This can be an electrical, chemical, optical or thermal signal [48].

There are different types of sensors that investigate different parameters, for instance the gas sensors. The progress of spoilage can be determined by the concentration of certain gases, like CO_2_ or H_2_S. The gas sensors make use of these properties by monitoring them. Those respond quantitatively and reversibly to the presence of a gas by changing the physical parameters of the sensor [10,49]. CO_2_ sensors are mostly non-dispersive infrared (NDIR) sensors or chemical sensors. NDIR sensors are spectroscopic sensors that measure the CO_2_ content by gas absorption at a certain wavelength [50]. Chemical CO_2_ sensors work with polymer or solid electrolytes. Infrared sensors as well as electrochemical, ultrasonic and laser technologies are used for the detection of O_2_ [36].

Another type of sensors are the biosensors. Compared to chemical sensors, they have a receptor made of biological materials such as enzymes, antigens, hormones, or nucleic acids. Depending on the measuring parameters, the transducer can be electrochemical, optical, acoustic, etc. For example, there is a biosensor (Toxin Guard by Toxin Alert) whose functional system is based on antibodies, which are integrated in plastic packaging and thus make it possible to detect pathogens such as *Salmonella*, *E. coli*, *Listeria*, and *Campylobacter*. A positive result is indicated by a visual signal [51]. Another biosensor is able to detect xanthine, which is an adenine nucleotide degradation product in animal tissue. To do this, xanthine oxide is immobilized on platinum, silver or pencil graphite electrodes [17].

#### 2.2.4. Other Systems

On the market there are already some other systems which have not been discussed in detail before. An overview of these systems is given in Table 3.

## 3. Advantages and Disadvantages of Intelligent Packaging Concepts

In general, intelligent packaging is easy to use and provide a number of advantages for consumers, food manufacturers, and the whole food industry. Depending on the system they offer different features [52]. 

The current quality status of a product can be determined by the use of indicators and sensors. This results in a general increase of product safety and in a reduction of unnecessary food waste [53,54]. In addition, this consistent quality monitoring also reduces time and material costs in the analysis methods of packaged food [55]. Further cost advantages also arise along the supply chain when intelligent packaging minimizes food waste. These aspects could be even more important in other life sciences like the pharmaceutical industry [54]. 

Data Carriers enable better traceability of the supply chain. Because of their low price, ease of use, and the benefit they provide, barcodes and QR Codes are nowadays widely spread. In contrast, indicators and sensors can be barely found on the market [13]. One reason for this is the price as the development and production costs are still very high [20]. The packaging costs can amount to 50–100% of the total costs of the final product. Actually a limit for packaging costs of 10% of the products value to be packaged is provided [56]. Furthermore, the use of indicators and sensors could lead to a negative change in consumer buying behavior: Customers would most likely put products with a discolored freshness indicator back on the shelf and choose a product with an uncolored freshness indicator. If the customer often sees labels of a brand product with a divergent color, he could even loose his confidence in that brand. At the same time, this behavior could also lead to an increase in unsold foodstuff [56]. On the other side, intelligent packaging can optimize the classic “first in—first out” principle. As the real current quality status of the food is known, the retailer can sell the products with the shorter shelf life first and so the wastage of food could be reduced [4].

It must be ensured that the systems are compatible with the food to be monitored. Not every intelligent packaging can be used for each type of food. Therefore, it must be clarified which indicator or sensor is appropriate for the product. The intelligent packaging can only be advantageous if it matches with the food. For instance, an oxygen sensor would be useful for MAP (Modified Atmosphere Packaging) packaged foods, while for chilled and frozen products a TTI should be apply [51].

Another aspect that still needs to be clarified is the recycling of the packaging. The additional waste generated by the installation and production of intelligent packaging is actually contradictory to the goal of reducing the amount of food wastage [55].

It should also be noted that it is not possible to rely a 100% on intelligent packaging for optimum product quality as misuse or failure of the systems cannot be ruled out [14]. Several factors are often responsible for the loss of quality. Monitoring just one parameter cannot provide a complete statement about the quality status of a product. Furthermore, external environmental influences such as light, temperature or mechanical stress can sometimes have an adverse effect on the technologies. On the one hand this can lead to a situation, where products are classified as no longer fit for consumption, even though they still are. On the other hand, this can result in a situation in which the spoilage of a product is not indicated. In the worst case, the consumer’s health may be adversely affected if the products are consumed. To sum up it can be said that the robustness of the systems must be improved and the individual packaging technologies should be combined in order to exploit as many advantages as possible [12,55].

## 4. Outlook and Future Perspectives

Due to changes in lifestyle, consumer demands or trends in commercialization, packaging has a major role in preserving fast moving consumer goods [39]. Unfortunately, intelligent packaging systems are not very widespread in the market yet. The reasons for this are the above-mentioned disadvantages (additional costs, acceptance of dealers/brand owners, etc.) of the systems. But the advantages of these systems should not be ignored [20]. Further research and improvement measures have to be conducted to use their benefits and enable a more extensive use [47]. 

**Figure 8:**
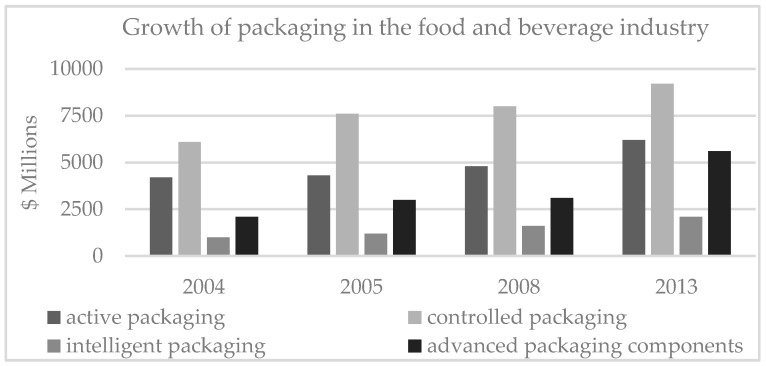
Growth of active, controlled, and intelligent packaging for the food and beverage industry 2004–2013 ($ millions), adapted from [19].

The interest in methods to improve food quality and safety as well as the management of the food supply chain is huge. There is a growing demand for the provision of information on packaging and food products. Consumers want to know which ingredients are in products or how the product was and should be stored [19,32]. Intelligent packaging has some advantages that could help to fulfill the aforementioned wishes [10]. This could lead to a slight increase in demand for these systems in the future (see Figure 8) [19].

However, an application is not meaningful or necessary for all areas. For each product type, it should be checked whether intelligent packaging is worthwhile or not. It only makes sense to use them if the use of these technologies increases sales or reduces waste, because intelligent packaging is associated with higher packaging costs [4]. The main areas of application should be perishable products such as meat or fish [4,57]. In the case of a very long shelf life or quality characteristics that can be easily identified by the customer such as the brown color of ripe bananas, no intelligent packaging is necessary [4].

Currently, intelligent packaging is mainly used in the food industry and rarely in the other life science industries although these systems may offer some advantages for these areas as well. They would also ensure a higher level of product safety in the pharmaceutical and cosmetics industries. As in the food sector, barcodes could ensure better traceability and temperature fluctuations could be determined using temperature indicators. 

As already mentioned, further research has to be done to enable a wider application [52]. Problems such as the price issue have to be solved [55]. Customers always want better quality and more information about the products, but most of them are not really willing to pay more for that. If they were well informed about the benefits of the systems, the customers might be more willing to spend more on food with intelligent packaging. In addition, consumer confidence in the safety of the systems also needs to be strengthened [15]. Therefore, further steps should be taken to promote the technologies [12]. Last but not least, the manufacturer also has to realize that the use of intelligent packaging can offer them a real market advantage. If all these aspects can be accomplished, a more extensive use of intelligent packaging would be possible.

## Figures and Tables

**Table 1:** Different examples and properties of commercially available TTIs (based on [36,37,38]).

Product Name	Principle	Color Range	Price Range (Cents)
**CheckPoint^®^ types M,L**	Enzymatic	Tricolor green to yellow to red	5–15
**Fresh-Check^®^**	Polymeric	Colorless to blue	1–5
**On Vu™**	Photochemical	Dark blue to colorless	1–5
**TT Sensor™**	Diffusion-reaction	Yellow to pink	5–15
**3M™ MonitorMark™**	Molecular diffusion	Colorless to blue	-
**eO**	Microbiological	Green to red	5–15
**Keep-it^®^**	Chemical reaction, based on immobile and mobile reactant	Movement of a blue bar to the left	-

**Table 2:** Principles of indicators and sensors based on metabolites (based on [36,41,42]).

Metabolites	Food Products	Indicators	Sensor
**Glucose/lactic acid**	Fermented food, meat	Colorimeter based on pH	Electrochemical sensor by redox reaction
**Carbon dioxide**	Fermented food, meat, seafood	Colorimeter based on pH	Electrochemical sensor by silicon-based polymers
**Oxygen**	Meat, vegetable, fruits	Optical sensor by fluorescence, colorimeter based on pH	Electrochemical sensor, laser
**Biogenic amines**	Fish, meat	Color-changing pH-sensitive dyes	Electrochemical sensor by enzyme redox reaction

**Table 3:** Types of commercially available intelligent packaging systems.

Technologies	Type	Name	Company
**Data carriers**	Radio frequency identification technologies	Easy2log Intelligent Box CS8304 Temptrip	CAEN RFID Srl Mondi Pic Convergence Systems Ltd. Temptrip LLC
**Indicators**	Time temperature indicators	CheckPoint^®^ Fresh-Check^®^ On Vu™ MonitorMark™ eO Keep-it^®^ Cook-Chex Timestrip Colour-Therm TopCryo™	Vitsab LifeLines Ciba Speciality Chemical and FreshPoint 3M™, Minnesota CRYOLOG S.A. Keep-it Technologies Pymah Corp. Timestrip Plc Colour-Therm TRACEO
	Freshness indicators	Fresh Tag SensorQ™ RipeSense Food fresh™	COX Technologies DSM NV and Food Quality Sensor RIpSense™ and ort Research Vanprob
**Sensors**	Bio sensors	Toxin Guard™ Food Sentinel^®^	Toxin Alert SIRA Technologies
	Gas sensors	Tell-Tab™ Ageless Eye™ O_2_Sense	IMPAK Corporation Mitsubishi Gas Chemical Inc. FreshPoint Lab.
